# Optical Property Stability of Light-Cured versus Precured CAD-CAM Composites

**DOI:** 10.1155/2022/2011864

**Published:** 2022-05-31

**Authors:** A. C. M. Andrade, A. B. Borges, E. C. Kukulka, S. E. Moecke, N. Scotti, A. Comba, C. R. Pucci, C. R. G. Torres

**Affiliations:** ^1^Department of Restorative Dentistry, Institute of Science and Technology, Sao Paulo State University—UNESP, Sao Jose Dos Campos, SP, Brazil; ^2^Department of Surgical Sciences, University of Turin, Dental School, Endodontics and Operative Dentistry, Turin, Italy

## Abstract

**Objective:**

The aim of this study was to evaluate the optical property changes after staining of precured (PC) and light-cured (LC) composites.

**Materials and Methods:**

Specimens were prepared using different LC composites (GrandioSO—Voco, Filtek Z350-3M/ESPE, Opallis—FGM, and Kalore—GC) and four PC blocks (Grandio Blocs—Voco, Lava Ultimate—3M ESPE, Brava Block—FGM, and Cerasmart—GC) from the same manufacturers (*n* = 20). Baseline color, gloss, translucency, and fluorescence were evaluated. The staining protocol was performed for 15 days, and the final optical properties were reevaluated.

**Results:**

The changes in each property were calculated (ΔGloss, ΔTranslucency, ΔFluorescency, Δ*E* *∗* 00). Data were analyzed by ANOVA and Tukey's test (*α* = 5%). Changes in all properties were observed after staining for all materials, with darkening and reduction of gloss, fluorescence, and translucency. Nonsignificant differences were observed between the light-cured and precured materials of the same manufacturer for Δ*G* and Δ*T*, but significant differences existed for Δ*F* and Δ*E* *∗* 00. For Δ*F*, the only significant differences were observed between Brava Block and Opallis (smaller). For Δ*E* *∗* _00_, only the light-cured composites GrandioSO and Z350 showed significantly less change than the corresponding blocks. Precured composites were affected the same way as light-cured ones by the staining in relation to the reduction of gloss and translucency.

**Conclusion:**

A higher reduction in fluorescence was observed for only one brand of block and was similar for the others. The two brands of light-cured materials showed less staining, while for the others, the staining was similar. The effects of staining vary according to the composite formulation.

## 1. Introduction

A frequent cause of composite restoration replacement is the change in their optical properties over time. These changes may lead to an unacceptable difference between the remaining tooth structure and the restoration [1]. The most important optical property concerning the esthetic outcome of composite restorations is color matching [2]. However, the proper translucency to simulate the enamel and dentin tissues [3] and a surface gloss similar to the neighboring enamel surface [4] are also very important. In addition, when exposed to an environment containing mainly UV light, the fluorescence of the restorative material in relation to the tooth is extremely relevant, and the fluorescence gives the restoration a bright and natural appearance, thus increasing its vitality [5, 6].

Adequate composite resin shade and translucency must be selected before the restoration is performed, allowing the creation of undetectable restorations [7]. However, the restorative material suffers different alterations in the oral environment over the years, resulting in loss of the desired optical characteristics [8]. Color alteration can occur inside or on the surface of the material. In the first case, it can be caused by chemical alteration of the composite matrix and polymerization initiators or by absorption of substances available in the oral environment [8–10]. In addition, insufficient polymerization causes inadequate irradiation from the light curing units and increases the residual monomer content, increasing water sorption as well as staining molecules [10, 11]. The use of higher concentrations of photoinitiator and tertiary amine in the material formulation can also increase color alteration [12].

The color change can also result from surface adsorption of different substances, which is mainly associated with the material surface roughness [13]. Some studies also showed that staining substances can produce degradation and softening of the composite polymer, reducing microhardness and increasing roughness [14–16]. A rougher surface loses its glossy aspect, affecting the esthetics of the restoration [17]. The softening of the organic matrix increases the wear exposure of inorganic fillers and their displacement, creating pores that increase biofilm accumulation and staining [18, 19]. Examples of these most common agents are coffee, wine, tobacco, ethanol, and different kinds of oils available in the regular human diet [20].

With the development and improvement of CAD/CAM technology, the use of precured composite blocks is becoming more popular [21, 22]. In this case, the polymerization of the material is performed by the industry using a chemical curing process under pressure and heating, which increases the degree of conversion and improves physical properties [23]. This improved polymerization is expected to increase mechanical properties and stability when exposed to the oral environment [24]. Studies have shown that polymerized composites outside the oral cavity, with additional pressures and different treatments, such as thermal treatment, can improve the physical properties and degree of conversion of composite resins, increasing restoration longevity [25].

In addition to the curing process, the composition of a composite may have great influence on the color stability of the material after exposure to different staining substances [26]. The most frequently used composites are the same as monomers in their organic composition: bisphenol A glycidyl methacrylate (Bis-GMA), triethylene glycol dimethacrylate (TEGDMA), dimethacrylate urethane (UDMA), and bisphenol A ethoxylated methacrylate, Bis-EMA [27].

Although the basic components are the same, each manufacturer chose a specific blend of resin monomers, which present different levels of water sorption that can interfere with composite staining. Studies showed that TEGDMA presented the highest level of water sorption, Bis-GMA and UDMA an intermediate level, and Bis-EMA the lowest level. One of the reasons was the presence of hydroxyl groups on Bis-GMA and UDMA, while Bis-EMA has a stiff central phenyl ring core [24, 27]. Another reason could be the lower degree of conversion and consequent unreacted residual monomers for certain blends [26]. The TEGDMA differences might be associated with their distinct physical structure [28]. The TEGDMA network is more heterogenic than the others, resulting in a larger microporous space between the polymeric agglomerates, which can be related to higher water sorption [27, 28]. Although there are no hydroxyl groups on TEGDMA, it presents water affinity because of the ether linkage binding structure, which is compatible with water [26]. Therefore, the ether linkages structures can increase the composite's water sorption.

Considering the differences between the conventional light-cured composites and the precured CAD-CAM blocks and their different formulations from various manufacturers, the aim of the present in vitro study was to evaluate the effect of staining substances, generally available on the oral cavity, over color, translucency, fluorescence, and gloss of different composite materials. The first null hypothesis tested was that the optical properties of conventional and CAD-CAM block composites do not differ from each other. The second and third null hypotheses were that the alteration of optical properties after immersion in staining solution is not related to the material (light-cured vs. precured CAD-CAM) or the composite configuration.

## 2. Materials and Methods

### 2.1. Specimens' Preparation

Cylindrical specimens 6 mm in diameter and 1.1 mm in height were prepared using four light-cured composites and four precured CAD-CAM blocks (*n* = 20). The technical information on the material tested is shown in Table 1. All materials were shade A2. For the light-cured ones, chromatic enamel shade was used, while for the composite blocks, the translucency level used was LT (low translucent).

The light-cured composite specimens were fabricated using a silicone matrix mold. The composite was applied in a single increment and light-cured for 20 s with an LED light-curing unit (Valo Cordless, Ultradent, Salt Lake City, Utah, USA) with a radiant emittance of 1000 mW/cm^2^. For the precured composites, cylinders were first obtained from the precured CAD-CAM blocks using a diamond trephine mill. To obtain specimens with the same thickness as the light-cured specimens, the cylinders were sliced using a diamond disc on a low-speed cutting machine (Labcut, Extec, Enfield, Connecticut, USA). The surface of all specimens was polished using silicon-carbide abrasive papers (grit #1200, #2400 and #4000, Extec Corp., Enfield, Connecticut, USA) in a polishing machine under water cooling for 30, 60, and 120 s, respectively. After polishing, all specimens were 1 mm in height.

### 2.2. Sample Size Calculation

The sample size calculation was performed using the G *∗* Power 4.11716 software (University Düsseldorf, Düsseldorf, Bundesland, Germany). First, the effect size 0.40 was determined. Considering a power of 95%, an error of 5%, and 8 experimental groups, a total sample size required was 144, with 18 per group. At the end, each group presented a total of 20 samples, for eventual loss of them, and for the safety of the study.

### 2.3. Color, Gloss, Translucency, and Fluorescence Measurements

Color and translucency measurements were performed by a colorimetric spectrophotometer (CM 2600d, Konica Minolta, Osaka, Kansai, Japan), adjusted for small area view (SAV), D65 standard illuminant, 100% UV included, observer angle of 2°, and specular component included (SCI). The reflectance data were converted to the chromatic coordinates L^*∗*^, a^*∗*^, and b^*∗*^, using the Spectramagic NX software (Konica Minolta, Osaka, Kansai, Japan). For analysis of translucency, the translucency parameter (TP) was calculated as the color difference between the L^*∗*^, a^*∗*^, b^*∗*^ coordinates obtained by placing the specimens over the white and black standard backgrounds [29, 30].

Gloss measurement was performed by a gloss meter device (Novo-Curve, Rhopoint, St Leonards-on-Sea, East Sussex, England), which presented a 2 × 2 mm reading area and 60° light incidence. The results were expressed in gloss units (GU). Three measurements were performed on each specimen, and the means of those measurements were used for statistical analysis [31, 32].

For the fluorescence, a spectrofluorophotometer (RF-5301 PC, Shimadzu Corp., Kyoto, Kansai, Japan) was used. This measurement was performed at a 365 nm wavelength for excitation [33] and a detection spectrum of 400–600 nm. The wavelength and intensity emission of each specimen were obtained.

### 2.4. Staining Protocol

A staining broth was prepared based on the American Dental Association (ADA) recommendation for laboratory testing, containing some common dental staining substances [34]. The broth was prepared with instant coffee, (Nescafe Classic, Nestle, Vevey, Riviera Vaudoise, Switzerland), black tee (Leao, Coca-Cola Company, Curitiba, Parana, Brazil), gastric mucin (Inlab, Diadema, Sao Paulo, Brazil), FD & C red (Cosmoquimica, Barueri, Sao Paulo, Brazil), FD & C yellow 5 red (Cosmoquimica, Barueri, Sao Paulo, Brazil), red wine (Santa Helena, Las Condes, Santiago, Chile), and distilled water. Specimens were immersed in the broth for 15 days at 37°C, with daily changes [35]. Then, the optical properties of the specimens were evaluated again.

The changes in gloss (Δ*G*), translucency (Δ*T*), and fluorescence (Δ*F*) were calculated by subtracting the final value from the respective baseline value. The color change was calculated using the Δ*E*^*∗*^_00_ formula, according to the Commission International L'Eclairage (CIE) [36].

### 2.5. Statistical Analyses

The normality of the data was evaluated by Shapiro-Wilk test, while the homogeneity of variances was analysed by Levene's test. To test the difference in optical properties between groups, both the baseline data and the data of changes in color (Δ*E*^*∗*^_00_), gloss (Δ*G*), translucency (Δ*T*), and fluorescence (Δ*F*) were submitted to one-way ANOVA and post hoc Tukey's test. For all analyses, a significance level of 5% was adopted, and Statistica for Windows software (StatSoft, version 9.1, Tulsa, Oklahoma, USA) was used.

## 3. Results

Mean values of gloss, fluorescence, and translucency obtained with different materials before immersion in staining broth, as well the results of one-way ANOVA and Tukey's test, are displayed in Table 2.

One-way ANOVA showed that GrandioSO, Opallis, and Brava Block are less glossy than the other tested materials, while Z350, Lava Ultimate, and Cerasmart showed the highest values. Regarding fluorescence, GrandioSO, Opallis, and Z350 are less fluorescent than all the others, which are significantly different among them. The composites Brava Blocks, Grandio Blocs, and GrandioSO are less translucent than all the others, while Kalore is the most translucent. When the gloss of light-cured and precured CAD-CAM materials from the same manufacturers was compared, Grandio Blocs showed higher values than the light-cured GrandioSO, while nonsignificant differences were observed for the other manufacturers. In relation to fluorescence, all blocks showed higher values than the light-cured version for all manufacturers. In relation to translucency, significant differences were observed only for FGM and GC manufacturers, with the CAD-CAM blocks being less translucent than the light-cured composites.

**Figure 1:**
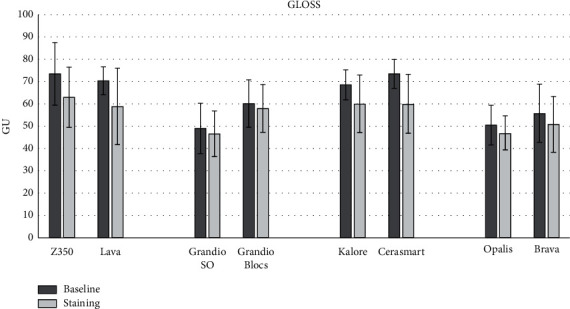
Means (SD) of gloss (GU) for all groups before and after staining.

**Figure 2:**
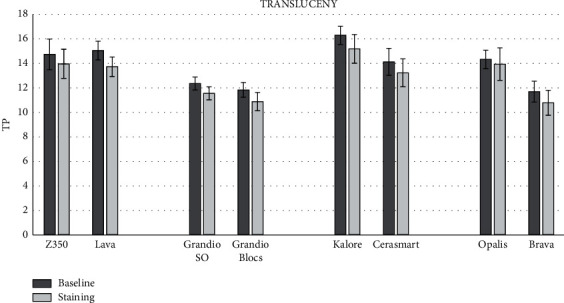
Means (SD) of translucency (TP) for all groups before and after staining.

**Figure 3:**
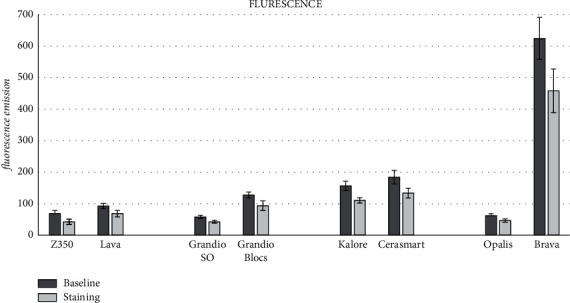
Means (SD) of fluorescence for all groups before and after staining.

The means for all optical parameters before and after staining are displayed in Figures 1–3, while the mean changes (±SD) in color (Δ*E*), gloss (Δ*G*), translucency (Δ*T*), and fluorescence (Δ*F*) are shown in Table 3. One-way ANOVA highlighted significant differences among the groups for the changes in translucency (Δ*T*) (*p*=0.0076), fluorescence (Δ*F*) (*p*=0.0001), and color (Δ*E*^*∗*^_00_) (*p*=0.0001). Nonsignificant differences were observed in the gloss parameter (Δ*G*) (*p*=0.0930). When the light-cured and precured CAD-CAM materials from the same manufacturers were compared, nonsignificant differences were observed for Δ*G* and Δ*T*. For Δ*F*, the only significant differences were observed between Brava Block and Opallis from FGM Company. For Δ*E*^*∗*^_00_, only the light-cured composites GrandioSO and Z350 showed significantly less change than the corresponding blocks.

Comparing all materials at the same time, nonsignificant differences were observed for Δ*G*. For Δ*T*, Opallis showed less change than Lava and Kalore. For Δ*F*, Opallis and GrandioSO showed less change than Cerasmart, while Brava showed a higher change than all the others. For Δ*E*^*∗*^_00_, Opallis and GrandioSO showed less change than Grandio Blocs, while Lava exhibited a higher color change than all the other materials.

## 4. Discussion

Composite resins are restorative materials used for esthetic procedures due to their optical properties being close to that of dental structure [37]. However, the color variability of human teeth justifies the need for composite systems that include different opacities and translucencies to allow the clinician to mimic the complex optical properties of enamel and dentin as much as possible. The optical properties of a restorative material are determined by several parameters, such as gloss, translucency, fluorescence, and color, which globally contribute to the overall appearance of a material.

The present study showed significant differences among the tested materials in relation to all optical properties evaluated, thus rejecting the first null hypothesis. Therefore, the clinician must consider those differences before performing a restoration to select the proper composite that best matches the optical characteristics of the involved teeth. For instance, in a clinical case where the dental enamel is highly translucent, materials with smaller translucency, such as GrandioSO, Grandio Blocs, and Brava Block (Table 2), would probably result in poor esthetics. On the other hand, a highly smooth labial surface of incisors, typical of middle- and old-age patients, would be better replicated with materials with higher gloss, such as Z350, Lava, Kalore, and Cerasmart.

Gloss and surface roughness are usually linked together, and the relationship between the two has been illustrated in previous studies [18, 38]. Tunac et al. [39] showed how filler size, distribution, geometry, and volume fraction could influence the polishing ability of composites, improving with smaller particle size and higher filler loading. The present study showed significant differences between light-cured composites, with GrandioSO and Opallis being less glossy than Z350 and Kalore. Z350 was the only pure nanoparticle light-cured composite tested in the present study, which could help explain the higher glossy surface when compared to other tested composites. On the other hand, Kalore contains prepolimerized silica filler, which can be easily worn by abrasive particles and produce a smooth surface [40].

Regarding CAD-CAM blocks, gloss was generally higher than light-cured counterparts even if only Grandio Blocs showed higher gloss than GrandioSO, which may be related either to its smaller filler content (3% less) or to the monomer combination (UDMA instead of Bis-EMA). The high-pressure/high polymerization process for CAD-CAM block fabrication should lead to a less porous material with a better filler distribution [22]. In addition, the manufacturing process led to a conversion degree of CAD-CAM blocks that is higher than that of the light-curing composites, which could affect polishing and final gloss [39]. Another possible explanation of the obtained results could be related to the monomer itself: generally, blocks contain UDMA monomer instead of Bis-EMA, which is present on the light-cured material. The Bis-EMA monomer previously presented a higher solubility level in comparison to UDMA, [24] probably due to the crosslinking pattern and the organic matrix strength or even being related to the filler-resin bond. Finally, although the kinds of fillers are the same, CAD-CAM blocks probably have a higher ratio of silicon dioxide nanoparticles in relation to the glass ceramic, but this information is not provided by the manufacturer.

Fluorescence, which was significantly higher in CAD-CAM blocks than light-cured composites of the same manufacturer, could impair the esthetics of a smile in an environment rich in UV light, such as in night clubs. The reason why the blocks from all manufacturers showed higher fluorescence than the light-cured materials is hard to explain. Most likely, the ingredient responsible for the fluorescence is higher in the blocks than in the light-cured materials, or the curing process can influence the fluorescence emission due to an unknown mechanism.

Translucency is one of the primary factors in evaluating dental esthetics [41]. The CAD-CAM blocks from FGM and GC showed significantly less translucency than the light-cured versions. This was expected, since the blocks are generally provided in two translucency levels, which are low translucency (LT) and high translucency (HT), and the one tested was the LT version. The blocks were compared with enamel shade composites, which are more translucent options for direct restoration. However, concerning the other manufacturers, the level of translucency of the LT version was similar to that of the direct enamel shades. Therefore, it is expected that the HT versions of those blocks would be higher than the enamel color of the corresponding direct materials.

Alterations of the optical properties over years are one of the reasons for restoration replacement, especially in the anterior teeth [20]. The present study results showed that the staining solution led to changes in optical properties, such as darkening, brightness reduction, fluorescence, and translucency. However, nonsignificant differences were found between light-cured and precured CAD-CAM blocks regarding Δ*G*, although significant differences were observed in Δ*T*, Δ*F*, and Δ*E*. Thus, the second null hypothesis was rejected. The composite staining can be related to their water sorption degree and to the resin matrix hydrophobic effect [9, 42]. If a composite absorbs water, it also absorbs other liquids, such as drinks with dark pigments that result in staining. The present study showed that esthetics alterations on composite do not occur for a single reason or to only certain types of composite. Liquid sorption of the resin matrix can lead to silane hydrolysis and microcrack formation, which allows penetration of the staining solution between the fillers and matrix, resulting in color change and consequent optical property alteration. This process could also impact the restoration outcome by expanding and plasticizing the resin component [43]. On the other hand, the reflectivity of light is related to the size of the filler and the filler-matrix homogeneity. The lower the filler-matrix homogeneity is, the lower the reflectivity of the light is [43]. Nonsignificant differences were observed for Δ*G*, even if a global reduction of gloss was observed in all tested materials, either light-cured or precured. The small size of the filler particles, which ensured that the light was scattered from the smooth surface, guaranteed gloss preservation, which was slightly altered by matrix dissolution, despite immersion in the staining solution.

Regarding Δ*T* results, the different amounts of inorganic components in some tested materials could impact the translucency worsening after immersion. Opallis showed less change than Lava and Kalore for Δ*T*: the resin with the least amount of inorganic content in its composition showed the least translucency changes, as stated by Salgado et al. [44]. In fact, Opallis presents an organic content of 78.5 to 79.5%, while Lava has 80% and Kalore has 82%. Lee et al. [45] analyzed the translucency parameters of some composites, and the results showed a change after storage in water. The composite resins absorb water at the matrix-filler interface and undergo hydrolytic degradation, changing the pattern of translucency and light diffusion. Although the difference in inorganic amount is small, it could help to explain the difference found between the tested composite resins.

The high-pressure/high polymerization process to which CAD-CAM blocks are submitted can also affect some changes in optical property, as observed in the present study, where the Δ*E* of Grandio blocs and Lava Ultimate had greater changes than their light-cured counterparts. Previous works showed that the refraction index of the composite matrix changes with the curing process [44, 45]. Gloss is a visual attribute of the geometric distribution of the reflected light from a surface and is an indicator of surface smoothness. The higher the gloss is, the higher the surface luster is, and it is known that a higher gloss can reduce the color difference effect, since the reflected light is predominant rather than the light reflected from the underlying composite material [46].

Based on the present study results, the third null hypothesis was rejected since the composite formulation did not influence the effect of staining on optical properties. Composites are exposed to the oral environment, saliva, food and beverage components, drugs and other external habits [9]. Water or liquid sorption occurs through absorption in the composite resin matrix [9]. Therefore, the water sorption amount depends on the filler content of the composite and the bond quality between the resin and filler. Another relevant point is that when excessive water sorption occurs, it decreases the composite quality by expanding and plasticizing the formulation components. The decrease in composite quality causes silane hydrolyzation and microcrack formation, which enhance composite staining and discoloration [9]. After immersion in staining broth, GrandioSO exhibited less color change than Grandio Blocs, while the precured Lava Ultimate compound exhibited the greatest color change. The polymeric matrix composition (BisGMA, UDMA, and TEGDMA) of these two types of resins may be the reason for this difference. The GrandioSO composite does not contain UDMA, which has been shown to be more resistant to staining than BisGMA. Under regular polymerization conditions, UDMA showed less water sorption than BisGMA, despite little difference [24], which might be the difference between the found results.

The present report evaluated gloss, translucency, fluorescence, and color. Measurements of the optical properties of composite resins, whether for direct or indirect use, after staining, are important for the possible esthetic variations that they may present in clinical situations. Despite this, recent research has shown that other variables can also be altered by acidic drinks and foods, such as hardness [47] and flexural properties [48], which were not considered in this study. Therefore, future in vitro tests are recommended, using other methods to simulate different responses to staining in the oral environment, as well as clinical trials are recommended.

## 5. Conclusions

It can be concluded that the staining process affected both the light-cured and precured CAD-CAM composites tested regarding gloss and translucency reduction. A higher fluorescence reduction was observed for only one brand of precured block and was similar to the others. For the two manufacturers, the light-cured materials showed less staining than the blocks, while for the others, it was similar. The staining effect varies according to the composite formulation.

## Figures and Tables

**Table 1:** Information about the composites tested.

Type	Name	Manufacturer	Classification	Composition^*∗*^
Light-cured	GrandioSO	VOCO GmbH, Cuxhaven, Cuxhaven, Germany	Nanohybrid	89% w/w of glass ceramic filler, functionalized silicon dioxide nanoparticles, pigments, Bis-GMA, Bis-EMA, TEGDMA, camphorquinone, butylated hydroxytoluene
	Filtek Z350	3M/ESPE company, Saint Paul, Minnesota, USA	Nanoparticle	78.5% w/w of silica nanoparticles, pigments, Bis-GMA, Bis-EMA, UDMA, and TEGDMA
	Opallis	FGM dental group, Joinville, Santa Catarina, Brazil	Nanohybrid	78,5% to 79,8% w/w of barium-aluminum silicate glass and silicon dioxide nanoparticles, camphorquinone, pigments, Bis-GMA, Bis-EMA, UDMA, and TEGDMA
	Kalore GC	GC Dental products, Tokyo, Island Honshu, Japan	Nanohybrid	82% w/w of glass fluoroaminosilicate, prepolymerized silica filler, silicon dioxide, DX-511, UDMA, dimethacrylate comonomers
Precured	Grandio Blocs	VOCO GmbH, Cuxhaven, Germany	Nanohybrid	86% w/w of and glass ceramic fillers, functionalized silicon dioxide nanoparticles, Bis-GMA, UDMA, TEGDMA
	Lava Ultimate	3M/ESPE company, Saint Paul, Minnesota, USA	Nanohybrid	80% w/w of zirconia nanoparticles, silica nanoparticles, UDMA, Bis-GMA, Bis-EMA, TEGDMA
	Brava Block	FGM dental group, Joinville, Santa Catarina, Brazil	Nanohybrid	65 to 80% barium glass, Bis-EMA, Bis-GMA, dimethylaminobenzoate, coiniciator, camphorquinone
	Cerasmart GC	GC dental products, Tokyo, Island Honshu, Japan	Nanohybrid	71% w/w of barium and silica nanoparticles, Bis-MEPP, UDMA, dimethacrylate

^*∗*^Bis-GMA; bisphenol A glycidyl methacrylate, TEGDMA; triethylene glycol dimethacrylate, Bis-EMA; bisphenol A ethoxylated dimethacrylate, UDMA; urethane dimethacrylate, DX-511; high molecular weight dupont monomer, and Bis-MEPP; bisphenol A ethoxylate dimethacrylate.

**Table 2:** Mean values (SD) and results of Tukey's test considering the absolute values of gloss, fluorescence, and translucency before staining.

Composite/manufacturer	Gloss^*∗∗*^	Fluorescence^*∗∗*^	Translucency^*∗∗*^
GrandioSO/Voco	49.03 (11.26) a	59.02 (3.72) a	12.38 (0.51) a
Grandio Blocs LT/Voco	60.07 (10.62) bc	128.47 (8.24) c	11.87 (0.55) a
Filtek Z350/3M ESPE	73.39 (14.20) d	70.38 (8.13) a	14.76 (1.22) bc
Lava Ultimate LT/3M ESPE	70.10 (6.50) d	95.58 (4.56) b	15.07 (0.78) c
Opallis/FGM	47.13 (7.73) a	63.42 (4.88) a	14.36 (0.75)bc
Brava Block LT/FGM	55.66 (13.11) ab	625.98 (65.74) f	11.71 (0.82) a
Kalore/GC	68.65 (6.58) cd	157.65 (14.08) d	16.33 (0.71) d
Cerasmart LT/GC	73.43 (6.53) d	185.85 (19.69) e	14.15 (1.05) b
ANOVA	0.000^*∗*^	0.000^*∗*^	0.000^*∗*^

^*∗*^Significant differences on the columns. ^*∗∗*^Groups followed by different letters on columns present significant differences.

**Table 3:** Means (SD) of color change and results of Tukey's test.

Composite	Δ*E*^*∗*^_00_	Δ*G*	Δ*T*	Δ*F*
GrandioSO-Voco	2.16 (1.12) a	−2.40 (10.50)a	−0.82 (0.52) ab	−16.14 (6.31) c
Grandio Blocs-Voco	3.39 (0.55) b	−2.13 (17.44) a	−0.96 (0.57) ab	−33.44 (16.03) bc
Filtek Z350-3M ESPE	2.69 (0.56) ab	−10.43 (11.97) a	−0.77 (0.34) ab	−26.99 (7.67) bc
Lava Ultimate-3M ESPE	5.15 (2.16) c	−11.23 (15.79) a	−1.30 (0.69) a	−25.58 (10.80) bc
Opallis-FGM	2.20 (0.95) a	−3.53 (6.53) a	−0.42 (1.09) b	−16.28 (7.25) c
Brava Block-FGM	2.51 (0.69) ab	−4.93 (14.32) a	−0.90 (0.37) ab	−167.11 (90.99) a
Kalore-GC	2.76 (1.47) ab	−8.63 (14.13) a	−1.09 (0.77) a	−45.97 (15.88) bc
Cerasmart-GC	2.95 (1.31) ab	−13.33 (12.60) a	−0.90 (0.65) ab	−50.48 (16.57) b
ANOVA	0.0001^*∗*^	0.0930	0.0076^*∗*^	0.0001^*∗*^

^*∗*^Significant differences on the columns.

## Data Availability

The data used to support the findings of this study are available from the corresponding author upon request.
